# Quantifying where human acquisition of antibiotic resistance occurs: a mathematical modelling study

**DOI:** 10.1186/s12916-018-1121-8

**Published:** 2018-08-23

**Authors:** Gwenan M. Knight, Céire Costelloe, Sarah R. Deeny, Luke S. P. Moore, Susan Hopkins, Alan P. Johnson, Julie V. Robotham, Alison H. Holmes

**Affiliations:** 1 National Institute of Health Research Health Protection Research Unit in Healthcare Associated Infections and Antimicrobial Resistance, Department of Infectious Diseases, Imperial College London, London, W12 0NN UK; 2 Data Analytics, The Health Foundation, London, UK; 3 Imperial College Healthcare NHS Trust, London, UK; 4 Antimicrobial Resistance Programme, Public Health England, London, UK; 5 Royal Free London NHS Foundation Trust Healthcare, London, UK; 6 Division of Healthcare-Associated Infection & Antimicrobial Resistance, National Infection Service, Public Health England, London, UK; 7 Modelling and Economics Unit, National Infection Service, Public Health England and Health Protection Research Unit in Modelling Methodology, London, UK

**Keywords:** Antibiotic resistance, Mathematical modelling, Community, Hospital, Resistance acquisition, Intervention design

## Abstract

**Background:**

Antibiotic-resistant bacteria (ARB) are selected by the use of antibiotics. The rational design of interventions to reduce levels of antibiotic resistance requires a greater understanding of how and where ARB are acquired. Our aim was to determine whether acquisition of ARB occurs more often in the community or hospital setting.

**Methods:**

We used a mathematical model of the natural history of ARB to estimate how many ARB were acquired in each of these two environments, as well as to determine key parameters for further investigation. To do this, we explored a range of realistic parameter combinations and considered a case study of parameters for an important subset of resistant strains in England.

**Results:**

If we consider all people with ARB in the total population (community and hospital), the majority, under most clinically derived parameter combinations, acquired their resistance in the community, despite higher levels of antibiotic use and transmission of ARB in the hospital. However, if we focus on just the hospital population, under most parameter combinations a greater proportion of this population acquired ARB in the hospital.

**Conclusions:**

It is likely that the majority of ARB are being acquired in the community, suggesting that efforts to reduce overall ARB carriage should focus on reducing antibiotic usage and transmission in the community setting. However, our framework highlights the need for better pathogen-specific data on antibiotic exposure, ARB clearance and transmission parameters, as well as the link between carriage of ARB and health impact. This is important to determine whether interventions should target total ARB carriage or hospital-acquired ARB carriage, as the latter often dominated in hospital populations.

**Electronic supplementary material:**

The online version of this article (10.1186/s12916-018-1121-8) contains supplementary material, which is available to authorized users.

## Background

Infections due to antibiotic-resistant bacteria (ARB) are associated with higher morbidity and mortality levels [1]. Globally, numbers of infections with ARB are increasing [2]. To tackle ARB, we need to develop interventions that optimise treatment outcomes whilst slowing the dissemination of antibiotic resistance. In order to develop these interventions we need to quantify the important transmission routes of ARB [3] and determine how much each setting contributes to the overall ARB burden. Without this information, we are potentially wasting resources on poorly targeted interventions [3, 4], resulting in delays in clinical care improvement and the continued spread of ARB to potentially irreversible levels [5].

ARB encountered in clinical situations may originate from any setting in which bacteria are exposed to antibiotics [6]. Such settings include hospitals, nursing homes, soil and wastewater from pharmaceutical plants [7, 8]. Although we know that antibiotics exist in many environments, we do not know what proportion of infections caused by ARB is due to the antibiotic exposure in each environment. For example, although a significant proportion of antibiotics is used in agriculture [9], there is an on-going debate about how much this usage selects for ARB that are ultimately transmitted to humans [10]. We therefore cannot currently predict the likely human health impact of reducing agricultural antibiotic use, although recent modelling work suggests that reducing transmission from livestock may be more important [11].

In this work we focus on two broad settings: the community and hospitals, as both are important for human ARB acquisition [12]. We define the “community” to be the population of individuals not in a healthcare setting. We did not include any settings with indirect pathways to human carriage of ARB, as the estimates are currently highly uncertain due to a lack of data (e.g. for agriculture [10]).

Although the vast majority (~ 80%) of healthcare antibiotics prescribed in England in 2013 were for patients in the community [13], the per capita exposure is greater and more infections with ARB occurred in hospitals [14]. This is worrying, as the hospital population suffer more serious consequences. What is unknown is whether, under a broad range of realistic parameters, ARB are commonly being acquired within the hospital setting or in the community and repeatedly introduced into hospitals within which they then spread. The former hypothesis suggests that antibiotic control in the community will do little to reduce the burden of serious ARB infections in hospitals, whilst the latter suggests that it may be key.

To address this unknown, we present a dynamic transmission mathematical model that tracks the acquisition of ARB by humans in each setting for a range of scenarios. The model structure is similar to previous modelling work exploring ARB movement between community and hospital settings (e.g. [12, 15–17]), but it is novel in that it generalises to multiple pathogen/antibiotic combinations to ask: Are there broad trends for acquisition that can be found, and under what parameter conditions are most ARB acquired in the community? Previous modelling work has focused on invasion of community strains into the hospital setting (e.g. [15, 18]) or mechanisms which drive maintenance of resistance in hospitals (e.g. [16, 19]). There is also a large body of work quantifying the different relative contribution of various colonisation or transmission routes of ARB in hospital wards [3, 4, 20–22]. This work expands on this quantification in the hospital, to explore the contribution of the hospital vs. community setting to acquisition of ARB under a large set of parameter combinations.

With this quantification we can explore whether broad trends exist using multiple scenarios, such as whether we would expect *most* ARB to be selected in the community or in the hospital and where interventions for ARB should be targeted. This adds to existing clinical data, which usually only report where the patient was when ARB carriage (asymptomatic or an infection) was detected but cannot differentiate where or how resistance was acquired: e.g. did a patient with a bloodstream infection in a hospital ward acquire that ARB in the hospital, or before, in the community? To link to a specific example, we considered the set of parameters for a case study of *Escherichia coli* resistant to third-generation cephalosporins in England, which are an increasing problem [23].

Our long-term aim is to quantify the sources of ARB [24]. With this study, we explore the relative contributions of two important environments and provide the basic structure to be expanded upon in future quantification work. This first step demonstrates what can and should be done for ARB research and also highlights the gaps in our existing understanding.

## Methods

**Fig. 1:**
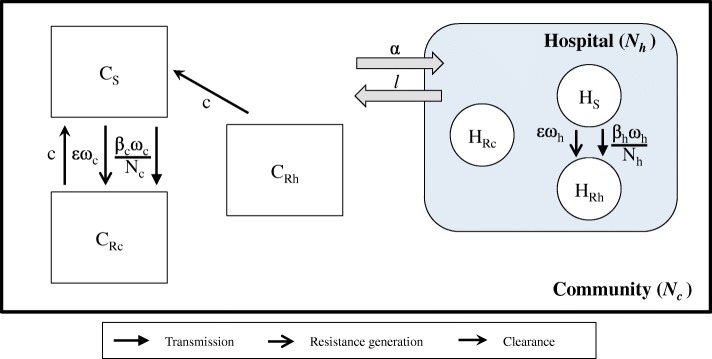
Model diagram of the community and hospital populations. Our compartmental model subdivides a human population into those in the community (*C*_X_) and those in the hospital (*H*_X_). People move between the hospital and the community (at rates α and *l*) and between further subpopulations depending on the ARB they carry and where they were acquired (X: S = susceptible, Rc = ARB acquired in the community, Rh = ARB acquired in the hospital). ARB acquisition is dependent on setting-specific transmission rates (β_c_, β_h_), antibiotic exposure levels (ω_c_, ω_h_) and population sizes (*N*_c_, *N*_h_) in the community or hospital respectively. ARB clearance occurs at a rate *c*

To determine where humans acquire ARB, we split a population of 100,000 people into subpopulations based on their setting (community or hospital) and bacterial status (with no bacteria or susceptible bacteria, or with ARB acquired within the hospital or community) (Fig. 1). Here “with bacteria” incorporates both carriage and infection. The community setting was taken to be broadly representative of the general population.

Within the population, people moved from the community (*C*_X_) into the hospital (*H*_X_) at the hospitalisation rate per day (α) and exited at a rate (*l*). Assuming that all hospitals are full [16], we initially set a constant percentage of 0.25% of the total population to be in hospital (but varied this in sensitivity analyses). People were grouped by their bacterial carriage status: carrying no bacteria or susceptible bacteria (*C*_s_, *H*_s_), carrying ARB acquired in the hospital (*C*_Rh_, *H*_Rh_) or carrying ARB acquired in the community (*C*_Rc_, *H*_Rc_). We did not differentiate people by age, gender, co-morbidity or colonisation/infection status. We assumed that infected and colonised people have the same infectivity [16, 25, 26], but that those “with” bacteria who become infected had a higher mortality rate [27].

ARB was acquired either by transmission at a rate (β_c_ω_c_/*N*_c_, β_h_ω_h_/*N*_h_) from an exogenous source or by de novo emergence (εω_c_, εω_h_) (Fig. 1). Here, β_c_, β_h_ are the transmission rates, ω_c_, ω_h_ the antibiotic exposure rates and *N*_c_, *N*_h_ the population sizes in the hospital and community respectively. ε is the proportion of people who acquire resistance during each antibiotic treatment. We chose a frequency-dependent transmission formulation to reflect likely transmission by the hands of healthcare workers in the hospital setting, where the likelihood of colonisation of the healthcare worker will depend on the proportion of patients carrying ARB, rather than the density. We used the same assumption in the community for consistency. Both acquisition rates (via transmission and de novo emergence) were dependent on exposure to antibiotics, as antibiotic use clears sensitive bacterial carriage, predisposing a host to colonisation with the (new) ARB. Linking transmission directly to antibiotic exposure captures this impact of selection on both the source of transmission (antibiotic exposure increases the ARB load) and the receiver (antibiotic exposure increases the chance of successful ARB transmission).

A higher transmission rate was (usually) assumed in the hospital (some models assume all transmission occurs only in hospitals, e.g. [16]). This is due to patient proximity, increased bacterial load and a high prevalence of immunocompromised patients in hospitals. The total number of contacts is likely to be greater in the community, due to the likely higher relative mobility of people in the community; however, each contact is likely to have a lower chance of successful bacterial transfer.

“Acquired” was defined by where the ARB were acquired, regardless of from whom they were transmitted. This is important in the public health context, as many infections with ARB are endogenous [28, 29], and hence knowledge of the original source of ARB acquisition is highly important for targeting interventions to reduce infections with ARB. The rate of transmission was taken to be a mass action assumption with random mixing in the hospital and community separately.

Those carrying bacteria can become infected (*i*_c_, *i*_h_) and die (μ_c_, μ_h_) at a higher rate than the background mortality rate. Due to potential fitness costs to resistant strains, it was assumed that resistant strains are equally or less likely to cause an infection than susceptible strains (by a factor *r*_inf_) [30]. People do not remain persistently colonised with ARB in the community, but instead carriage is lost at a rate *c*. Due to the short duration of stay and the likely higher transmission rates in hospital, it was assumed that resistant bacterial carriage is not lost in the hospital setting.

These bacterial and patient dynamics (Fig. 1) were captured using a compartmental, deterministic model (see Additional file 1). All parameter values are listed in Table 1. Our results, as they are proportions, remain the same for any large population size. Detailed explanations of methods to determine the ranges for each parameter are given in Additional file 1.

**Table 1:** Parameter values with description and range of parameters explored as well as the values used in the case study. For all details on calculations see Additional file 1

Symbol	Parameter description	Range	Case study	Notes and references
*N*	Total population size	100,000	100,000	Fixed
*N*_h_	Size of the total population in hospital	(0.02% to 3%)*N*	0.25%	Fixed in baseline [37], explored in sensitivity analysis
*N*_c_	Size of the total population in the community	(1 – [0.02% to 3%])*N*	1–0.25%	Depends on *N*_h_
α	Rate at which those in the community enter the hospital	2 × 10^−4^ to 2 × 10^−3^ per day	8 × 10^− 4^ per day	Linked to number of admissions per day [38]
*l*	Rate at which those hospitalised return to the community	0.05 to 1 per day	0.32 per day	Varied to fit *N*_h_
*b*	Background death rate	Fixed	1/(81*365)	Inverse of life expectancy [39]
ε	Proportion that acquire resistance during each antibiotic treatment	0.0008 to 0.13	0.0135 per treatment	Estimates taken from a range of studies (see Additional file 1)
ω_c_	Rate of antibiotic exposure in community	(1 to 15)/1000 per day	8.6/1000 per day	Using total consumption in England in 2014 [23] and point prevalence surveillance data [40]
ω_h_	Rate of antibiotic exposure in hospital	(0.5 to 1.00)ω_c_	0·22 per day	
β_h_	Transmission rate in the hospital	0.1 to 10 per day	1.8 per day	Case study value calibrated [14, 41]. Assumed to be the same or lower in the community
β_c_	Transmission rate in the community	β_h_/25 to 2β_h_	β_h_	
*c*	Rate of clearance of resistant bacteria in community	1/730 to 1/42 per day	1/127 per day	Estimates taken from a range of studies (see Additional file 1)
*i*_c_	Rate of infection in the community	(1.4 to 2.8) × 10^− 6^	1.75 × 10^− 6^	[42]
*i*_h_	Rate of infection in the hospital	(5 to 500)*i*_c_	100*i*_c_	Assumed to be higher in hospitals due to patient co-morbidities.
*r*_inf_	Decreased rate of infection by resistant organisms	0.5 to 1	0.8	Most ARB have reduced fitness, which can be ameliorated. (see Additional file 1)
μ_r_	Proportion of infections with resistant bacteria that result in death	0.4 to 0.9	0.6	Case study value based on bacteraemia data [27]
μ_c_	Proportion of infections with susceptible bacteria that result in death	0.1 to 0·5	0.2	

### Case study

To give a specific parameter combination, we considered a case study of *E. coli* in England, focusing on phenotypic resistance to third-generation cephalosporins, which is commonly, but not exclusively, mediated by production of extended-spectrum β-lactamases (ESBLs). We chose this due to the frequent use of β-lactams in both community and hospital settings, the existence of mandatory surveillance data for *E. coli* bacteraemia and due to the increasing problem of resistance (see Additional file 1) [23].

### Total population analysis

Our primary outcome measure was the proportion of the total population with resistance who had acquired it in the hospital (Eq. (1)).

1 $$ Proportion\ of\ resistance\ in\ total\ population\ acquired\ in\ hospital=\frac{H_{Rh}+{C}_{Rh}}{H_{Rc}+{H}_{Rh}+{C}_{Rc}+{C}_{Rh}} $$

Due to the high levels of parameter uncertainty, our results are presented across many parameter combinations to encompass many possible resistance types. Firstly, we performed bivariate parameter analysis, with all other parameters held at the values in the case study. Secondly, in order to further explore multivariate effects, we used Latin hypercube sampling (LHS) (a method by which a well-distributed set of parameters is generated from a multidimensional distribution) to generate 10,000 parameter sets from our parameter ranges (Table 1). These 10,000 parameter sets allowed us to explore, within reasonable bounds, many possible combinations of values for each of the clinical variables in the model, for example, high antibiotic usage in hospitals with low lengths of patient stay and vice versa. Extreme parameter combinations resulted in negative population sizes, which arose due to our use of a discrete-time simulation. We removed these values, to leave our final valid parameter set. With the variation in exit and entry rates, the size of our “hospital” population varied from 0 to 4% of the total population. This could reflect a larger hospital population than is currently the case for England, or our “hospital” setting representing a hospital population plus other populations with similar characteristics (e.g. high antibiotic exposure) such as nursing homes, where approximately 0.5% of the English population resides [31].

Using the valid LHS parameter samples we performed a sensitivity analysis to consider which parameters drive relative acquisition and hence should be targeted for both interventions and further data collection. This was done using a partial rank correlation coefficient analysis (PRCC) [32].

### Hospital population analysis

The preceding multivariate analysis was repeated for the hospital subpopulation with a similar outcome measure: what proportion of those with ARB in hospital had acquired it in the hospital setting (Eq. (2)). This allowed us to explore whether those in the hospital setting have a different place of ARB acquisition than the total population.

2 $$ Proportion\ of\ resistance\ in\ hospital\ population\ acquired\ in\ hospital=\frac{H_{Rh}}{H_{Rc}+{H}_{Rh}} $$

A sensitivity analysis was also performed for this outcome (Eq. (2)) and for the prevalence of resistance in the population.

## Results

### Analysis of the total population

**Fig. 2:**
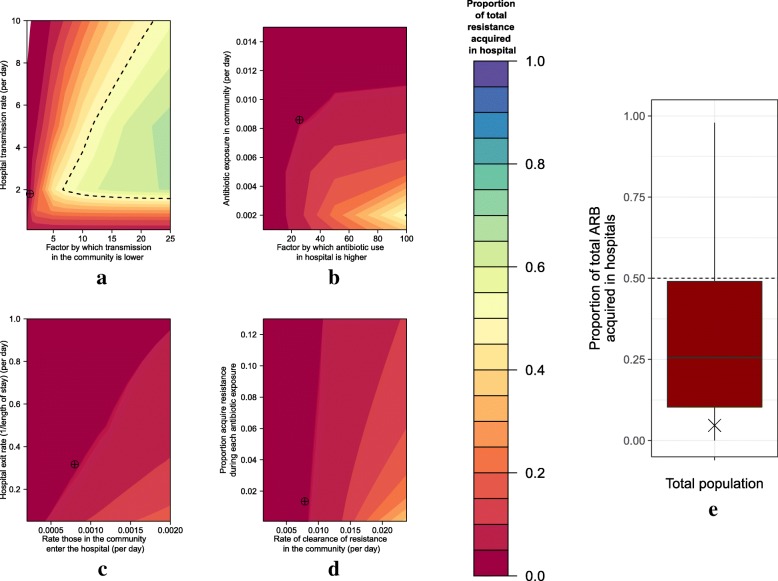
The proportion of the total population with resistance that was acquired in the hospital at different parameter values. Here, *red shading* indicates that the minority in the total population acquired resistance in the hospital setting under bivariate (**a**–**d**) and multivariate parameter analysis (**e**). The *dashed lines* indicate the boundary of 50% resistance acquired in the hospital. *Blue/green shaded areas* indicate parameter combinations where the majority of human acquisition was in the hospital setting. The bivariate parameter combinations were of **a** varying transmission rates, **b** varying antibiotic exposure rates, **c** varying entry and exit rates into the hospital and **d** varying clearance and acquisition rates. Note that for **a**–**d** all other parameters are held at their case study values. **e** Most people with ARB in the total population had acquired ARB in the community setting for the majority of our LHS parameter samples. The targets (**a**–**d**) and cross (**e**) indicate the parameter combinations in our case study

The minority of human ARB acquisition in our case study of *E. coli* resistant to third-generation cephalosporins occurs in the hospital (5%) (targets in Fig. 2).

Under most of our parameter combinations (bivariate and multivariate) the majority of the ARB in the total population was acquired in the community (Fig. 2). Only under certain scenarios was more resistance acquired in the hospital (Fig. 2, green to blue shaded areas above the 50% cut-off dashed line). Importantly, if the rate of transmission in the hospital is increased (*y* axis, Fig. 2a), then acquisition in the hospital is greater when the level of transmission in the community is lower by a factor of six or more (we explored up to a factor of 25 times lower). At extremely low transmission levels, more resistance is acquired in the community setting, due to a reversal to de novo resistance generation (instead of transmission) domination in the larger community population.

Even when antibiotic exposure is much higher in the hospital than in the community setting, acquisition in the hospital does not dominate in our model (Fig. 2b). Similarly, even when the exit rate from hospital is extremely low (i.e. there are long lengths of stay in hospital) or the rates of clearance of resistance in the community are high, acquisition in the hospital does not dominate (Fig. 2c–d). Varying the infection or mortality rates had little impact on these results (see Additional file 1).

More of the total ARB burden was acquired in the community under the majority (76%) of the valid (6562) LHS parameter sets (Fig. 2e). The proportion of acquisition in the community varied by parameter set, with a mean of 69% of ARB acquired in the community (see Additional file 1). Similar results were seen across different hospital population sizes (see Additional file 1).

**Fig. 3:**
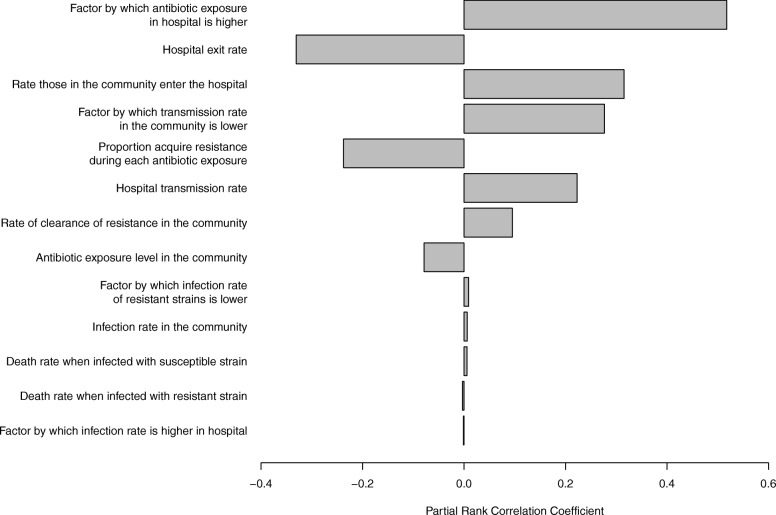
Tornado diagram of the key drivers of resistance acquisition in hospitals from partial rank correlation coefficient analysis. The parameters with the highest absolute values have the greatest influence on the proportion of resistance in the total population acquired in hospital

Our sensitivity analysis showed that the most influential parameters on the proportion of ARB acquired in the hospital are those of relative antibiotic exposure, exit/entry rates and transmission in the hospital (Fig. 3).

### Analysis of the hospital population

**Fig. 4:**
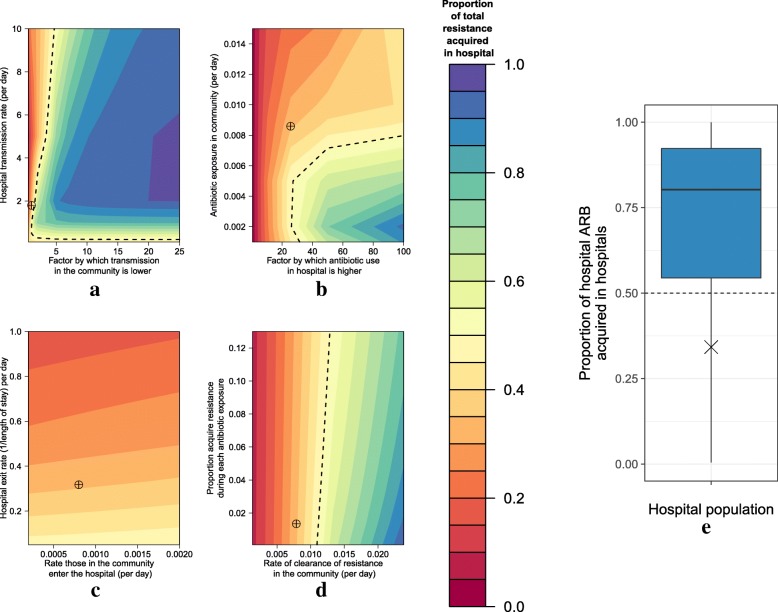
The proportion of the hospital population with resistance that was acquired in the hospital at different parameter values. Here, *red shading* indicates that the minority in the hospital population acquired resistance in the hospital setting under bivariate (**a**–**d**) and multivariate parameter analysis (**e**). The *dashed lines* indicate the boundary of 50% resistance acquired in the hospital. *Blue/green shaded areas* indicate parameter combinations where the majority of human acquisition was in the hospital setting. The bivariate parameter combinations were of **a** varying transmission rates, **b** varying antibiotic exposure rates, **c** varying entry and exit rates into the hospital and **d** varying clearance and acquisition rates. Note that for **a**–**d** all other parameters are held at their case study values. **e** Most people with ARB in the hospital population had acquired ARB in the hospital setting for the majority of our LHS parameter samples. The targets (**a**–**d**) and cross (**e**) indicate the parameter combinations in our case study

In the hospital population, the minority of human ARB acquisition in our case study of *E. coli* resistant to third-generation cephalosporins also occurs in the hospital (35%) (targets in Fig. 4). This was an exception to the majority of parameter scenarios, where most patients with ARB in hospital had acquired this resistance whilst in the hospital setting (Fig. 4). The exceptions were when transmission in the hospital was high, and similar in the community (Fig. 4a); when there was high antibiotic use in the community or similar levels in both settings (Fig. 4b); and when the rate of clearance in the community was low (Fig. 4d). Varying the exit and entry rates from those assumed in our case study (Fig. 4c) also generated no scenarios where most ARB in the hospital setting were acquired in the hospital setting.

When considering only those with ARB in the hospital, more had acquired them in the hospital under the majority (78%) of the valid LHS parameter samples (Fig. 4e). The proportion acquired in hospital varied across parameter sets, with a mean of 71% of ARB acquired in hospitals (see Additional file 1).

Our sensitivity analysis showed that the most influential parameters on the proportion of ARB acquired in the hospital for those patients in hospital are those of antibiotic exposure, rate of clearance of resistance in the community and length of stay (hospital exit rate) (see Additional file 1). For overall prevalence of resistance (see Additional file 1), the rate at which resistance is cleared and antibiotic exposure in the community, as well as the proportion that acquire resistance during antibiotic exposure are the most important parameters.

## Discussion

Our work suggests that most ARB in the total population are acquired in the community setting. If instead we consider the small hospital subpopulation, under the majority of parameters considered, those patients in hospital with ARB had acquired them in the hospital. Quantitative assessment frameworks such as this can be used to make much sought-after predictions regarding the spread of ARB and the impact of interventions [7]. For example, this output forms part of the evidence base required for the recommended interventions, such as where to prioritise vaccine or diagnostic rollout, in the recent UK O’Neill Report on “tackling drug-resistant infections globally” [33]. Such information is urgently needed, as ARB is already a major societal issue globally [2, 10].

The key parameters that alter where resistance is acquired are antibiotic use, length of hospital stay and the rate of transmission of ARB. Only under scenarios of much greater levels of transmission (Fig. 2a, upper right-hand corner) or antibiotic use in hospitals (Fig. 2b, lower right-hand side) is human ARB acquisition in the total population driven by hospitals. The predominance of human acquisition of ARB in the community is linked to the substantially higher numbers of people in the community (~ 98% of our population). If we increase the percentage of the population in our “hospital” setting, then the proportion acquired in hospitals increases, as seen by Kouyos et al. when exploring hospital size and ARB [17]. It is then crucial for intervention design and our understanding of ARB that we know the details of the heterogeneous settings in our populations and their interrelationships.

If reducing the total acquisition of ARB is our goal, then this model suggests interventions should target antibiotic exposure in the community setting. There are many ways that this could be done, for example by using educational interventions [34] or by targeting the symptoms most likely to be inappropriately prescribed antibiotics, such as sore throat [35]. Within the hospital setting, this model suggests that to reduce acquisition of ARB here, interventions should target transmission (for example by improved hand hygiene) and reduced antibiotic exposure. More acquisition also occurs in the hospital setting if clearance rates are higher in the community, suggesting that post-discharge decolonisation regimes, whilst aiding in driving down resistance prevalence, may shift the majority of ARB acquisition from occurring in the community to the hospital setting. Length of stay was also an important driver of where acquisition occurs, which could be targeted by tackling fundamental infrastructure (e.g. improved outpatient care).

To understand the clinical implications of this work we also need to consider the following question: does a reduction in human acquisition of ARB, which we model here, directly lead to a decrease in their associated health burden? We found that to reduce total ARB carriage requires interventions against acquisition of ARB in the community. However, the majority of those with ARB in hospitals had acquired them in the hospital. Although the hospital population is very small (< 4% of the total population), it is the one in which infection with ARB is potentially far more serious due to the higher proportion of people with immunocompromised status. Hence, it could be argued that reducing ARB burden in hospitals would have a bigger health impact. Targeting ARB in hospitals may also have a knock-on effect if those in hospital are the key sources of on-going transmission due to their immunocompromised status and increased bacterial load. Thus, the link between ARB acquisition and impact on health burden needs to be determined. Similarly, the routes to successful acquisition need to be established. For example, in exploring antibiotic use in agriculture, what proportion of those who eat meat contaminated with ARB subsequently become infected?

Our case study highlights that our choice of where we target interventions should be tailored by the type of resistance and pathogen under consideration. Here, for *E. coli* resistant to third-generation cephalosporins, this work suggests that interventions should be focused on the community setting, as the majority of ARB acquisition (even in the hospital population) was always in the community (crosses in Figs. 2e and 4e). This reflects the parameters of this case study, where we assumed that transmission rates were the same in the hospital and community, that acquisition rates per treatment were low and, importantly, that high levels of cephalosporins are used in the community. For other ARB with high levels of antibiotic exposure in the community (such as other β-lactams) it may be that most acquisition is always in the community setting. However, for the majority of our parameter combinations, and hence other ARB, the picture is more complex, and the levels of use of the specific antibiotic in each setting will be critically important in determining where acquisition occurs. This can be seen through the dependence of our results on antibiotic exposure in the sensitivity analysis (Fig. 3).

The strengths of our study are that it uses a transparent quantitative framework to explore a broad parameter range that encompasses a wide set of potential scenarios for ARB acquisition. As there are few good estimates for many of these parameters (e.g. transmission rates), this allows for only broad conclusions to be drawn. Moreover, this model captures only a subset of the dynamics — both important human population and environmental stratifications are missing. Including missing human population stratifications (e.g. age, colonisation and infection status, and co-morbidities) would alter the movements between settings, requiring lengths of stay and contact pattern distributions, antibiotic exposure rates, as well as mortality rates. In particular, it is known that resistance prevalence is highest in those with longer lengths of hospital stay. Environmental stratifications could include agricultural and waste water contact.

In comparison to previous work, our analysis is novel in that it considers acquisition of ARB from a broader, more general quantitative perspective, namely: how much ARB is acquired in which setting? This differs from previous mathematical models of ARB spread in the community and hospital [12, 16, 17, 25] which consider the importance of community reservoirs and the contribution of incoming carriage rates to their primary focus of the hospital.

The parameter sensitivity analysis suggests that future work should focus on determining better estimates for levels of transmission, antibiotic exposure and the rate at which ARB are cleared. Their correlation with the proportion of resistance acquired in the hospital suggests that these parameters are also key targets for interventions.

The next steps for this model would be to include the additional structural complexities that can be parameterised. Specifically, the entry and exit rates for the population should be stratified, potentially by age, as these rates were found to be key influences on the proportion of resistance acquired in hospitals. This lack of heterogeneity is the main limitation of this model. Including further human population stratification would likely result in acquisition differences by age (e.g. more acquisition in the community in certain age groups than others). The model could also be tailored to suit different pathogens and resistance types, as the quantitative contributions of different environments are likely to vary. One assumption to be varied is the 100% carriage rate, which is not true for pathogens such as *Staphylococcus aureus* and its important resistant subpopulation (methicillin-resistant *S. aureus*, MRSA). Complexity here would need to be added in the form of assumptions around the protective effect of prior colonisation.

Strikingly, most antibiotic resistance modelling studies tackle only populations in the community, hospitals, families and schools [36]. Extending our framework, for example to build on previous work showing the likely contribution of livestock antibiotic usage [11], could be used to test hypotheses, evaluate trends in resistance development over time and test the relative impact of new interventions. Furthermore, if we could extend our model to quantify how much ARB acquisition takes place in different hospital settings, such as wards, then we could potentially design better treatment options (e.g. if little ARB acquisition occurs in intensive care units, then last line antibiotics could be used there for critically ill patients).

To test our model results would require new data to be collected; for example, there could be a large prospective longitudinal study, tracking where and how people acquire ARB. This could be done by routinely sampling an individual’s microbiome and that of his/her environment, to determine when (if at all) and where (community or hospital) acquisition of ARB occurs. The study would have to be large and long (~years) to be powered to detect hospital vs. community differences due to the low rate of hospitalisation and low prevalence of ARB. In the absence of such a large trial, efforts to determine differences in parameters, such as average antibiotic exposure levels in community and hospital settings, could be used to improve our parameter estimates. This would narrow the range of parameters explored within this model and allow us to be more confident as to where acquisition is occurring. Our model predictions of relative levels of acquisition at these new estimates could then be tested by targeting interventions either at the community or hospital (whichever the model deems to be the setting of most acquisition) and comparing impact on ARB carriage and infection levels.

This study provides a new framework for ARB source quantification. We need now to not focus solely on where ARB infections are detected but on the settings where ARB are acquired. With this knowledge we can target ARB acquisition at its source, rather than fire fight at the clinical endpoint. More research is crucially needed on ARB prevalence across healthcare and environmental settings, transmission routes for ARB that result in human health burden and the levels and acquisition effects of antibiotic usage.

## Conclusions

This is the first step to building a quantitative framework to test the relative contributions of all of the complex multidimensional drivers of ARB acquisition and hence improve intervention design. Here, we highlight the complex relationships that are likely to be uncovered by showing that, under the majority of our resistance scenarios, although the majority of ARB acquisition occurs in the community, most people with ARB in the hospital have acquired it in the hospital setting. Future work needs to develop this model to capture the full spectrum of ARB sources and to capture data on acquisition and transmission across these source environments.

## Additional file

Additional file 1: Additional information: Model equations and details of parameterisation. Additional results: Proportion of the population in hospital, variation in infection and mortality rates, histogram of parameter sets, sensitivity analyses. (PDF 177 kb)

## Abbreviations

ARB: Antibiotic-resistant bacteria
ESBL: Extended-spectrum β-lactamase
LHS: Latin hypercube sampling
MRSA: Methicillin-resistant *Staphylococcus aureus*
PRCC: Partial rank correlation coefficient analysis
