# Supplementary material for: Quantifying where human acquisition of antibiotic resistance occurs: a mathematical modelling study
Source: BMC Med. 2018 Aug 23;16:137. doi: 10.1186/s12916-018-1121-8 (PMC6106940; doi:10.1186/s12916-018-1121-8)
Supplement: Supplementary file 1 — Additional information: Model equations and details of parameterisation. Additional results: Proportion of the population in hospital, variation in infection and mortality rates, histogram of parameter sets, sensitivity analyses. (PDF 177 kb) [file 12916_2018_1121_MOESM1_ESM.pdf]

Supplementary material for: A First Step to Quantifying Where Human Acquisition of Antibiotic Resistance Occurs: A mathematical modelling study

Gwenan M. Knight[1*], Ceire Costelloe[1], Sarah Deeny[2], Luke S. P. Moore[1,3], Susan Hopkins[1,4,5], Alan P. Johnson[1,6], Julie Robotham[1,7], Alison H. Holmes[1,3],

1 National Institute of Health Research Health Protection Research Unit in Healthcare Associated Infection and Antimicrobial Resistance and Department of Infectious Diseases, Imperial College London, London, UK
2 Data Analytics Team, The Health Foundation, London, UK
3 Imperial College Healthcare NHS Trust, London, UK
4 Antimicrobial Resistance Programme, Public Health England, London, UK
5 Royal Free London NHS Foundation Trust Healthcare, London, UK
6 Healthcare Associated Infections and Antimicrobial Resistance, Public Health England, London, UK
7 Modelling and Economics Unit, Centre for Infectious Disease Surveillance and Control, Public Health England and Health Protection Research Unit in Modelling Methodology, London, UK

Contents

1 Model equations and details **2**
1.1 Model equations 2
1.2 Detailed model dynamics 2
1.3 Model simulation 3

2 Parameter calculations **3**

3 Additional results **7**
3.1 Proportion of the population in hospital 7
3.2 Variation in infection and mortality rates 8
3.3 Histogram of parameter sets 9
3.4 Sensitivity analysis for hospital population 9
3.5 Sensitivity analysis for resistance prevalence 9

1 Model equations and details

1.1 Model equations

Let C_s be the number of people with susceptible bacteria in the community and H_s the equivalent number in hospital. Let C_{Rc} be the number with resistant bacteria in the community who acquired the resistant bacteria (either via transmission or *de novo*) in the community and let the number in the community with resistant bacteria acquired in hospital be C_{Rh}. The equivalent populations in the hospital are H_{Rc} and H_{Rh}. The status of everyone carrying bacteria is appropriate for a commensal such as *E. coli*. These sub-populations interact via the dynamics shown in the difference equations in (1).

$$
\begin{aligned}
C_s[i+1] &= C_s[i] + lH_s[i] - (\alpha + \omega_c\epsilon)C_s[i] - \beta_c\omega_c(C_{Rc}[i] + C_{Rh}[i])C_s[i]/Nc \\
&\quad + c(C_{Rc}[i] + C_{Rh}[i]) + i_{cr}\mu_r(C_{Rc}[i] + C_{Rh}[i]) + i_{hr}\mu_r(H_{Rc}[i] + H_{Rh}[i]) + i_h\mu_s H_s[i] \\
&\quad + b(C_{Rc}[i] + C_{Rh}[i] + H_s[i] + H_{Rc}[i] + H_{Rh}[i]) \\
C_{Rc}[i+1] &= C_{Rc}[i] + lH_{Rc}[i] - \alpha C_{Rc}[i] + \omega_c\epsilon C_s[i] \\
&\quad + \beta_c\omega_c(C_{Rc}[i] + C_{Rh}[i])C_s[i]/Nc - (c + i_{cr}\mu_r)C_{Rc}[i] - bC_{Rc}[i] \\
C_{Rh}[i+1] &= C_{Rh}[i] + lH_{rh}[i] - \alpha C_{Rh}[i] - (c + i_{cr}\mu_r + b)C_{Rh}[i] \\
H_s[i+1] &= H_s[i] - lH_s[i] + \alpha C_s[i] - \omega_h\epsilon H_s[i] - \beta_h\omega_h(H_{Rc}[i] + H_{Rh}[i])H_s[i]/Nh \\
&\quad - (i_h\mu_s + b)H_s[i] \\
H_{Rc}[i+1] &= H_{Rc}[i] - lH_{Rc}[i] + \alpha C_{Rc}[i] - (i_{hr}\mu_r + b)H_{Rc}[i] \\
H_{Rh}[i+1] &= H_{Rh}[i] - lH_{Rh}[i] + \alpha C_{Rh}[i] + \omega_h\epsilon H_s[i] \\
&\quad + \beta_h\omega_h(H_{Rc}[i] + H_{Rh}[i])H_s[i]/Nh - (i_{hr}\mu_r + b)H_{Rh}[i]
\end{aligned}
\tag{1}
$$

1.2 Detailed model dynamics

Patients move from the community into the hospital at a rate (α) which is equal to the hospitalization rate per day (Table 1). The reverse, movement of people from the hospital into the community (l) is the inverse of the length of hospital stay. This exit rate was varied to maintain the desired proportion of the population in the hospital in each scenario. It is assumed that all people, whether carrying susceptible or resistant bacteria have the same characteristics in terms of entry and exit. In our case study, the required constant length of stay to give $0{\cdot}25\%$ of the population in hospital was $3{\cdot}15$ days (Table 1).

People can acquire ARB either by transmission or by *de novo* emergence. The latter is determined by assuming that a proportion (ϵ) of every person that is treated with (or exposed to) antibiotics (at a rate ω) becomes dominantly colonised by ARB. It is difficult to determine estimates for this proportion (ϵ) but it is assumed to be the same for hospital and community acquisition so should have little impact on the relative results.

The antibiotic exposure parameter (ω, the probability of being given an antibiotic per day) differs by setting and is assumed to (usually) be higher in hospitals than in the community. Within the parameter variation, we also explored simulations under which treatment rates with antibiotics in the community exceed those in the hospital. In the case study, it is assumed that 22% of inpatients on any given day are receiving beta-lactam antibiotics and that $0{\cdot}86\%$ of those in the community are [1, 2] (Table 1).

The rate of transmission is taken to be a mass action assumption (the more people carrying bacteria, the more secondary cases) with random mixing in the hospital and community separately. The number

of new cases with resistance is the transmission parameter (β_h, β_c for hospital or community respectively) multiplied by the number of people with susceptible bacteria, the proportion of the population with resistant bacteria and the level of antibiotic exposure (all in the relevant setting). This transmission parameter (or effective contact rate) is a product of the number of contacts per day and the probability that transmission of bacteria occurs in that contact. It was assumed that this rate differs between the settings - there are assumed to be usually be the same or more effective transmission events in the hospital setting. This is similar to assumptions in previous work considering transmission of other resistant bacteria: for a meticillin-resistant *Staphylocccus aureus* (MRSA) model of spread in hospitals and the community it was assumed that transmission only occurs in hospitals [3]. This is because in hospitals it is potentially more likely that successful transmission of bacteria will occur due to proximity of patients, increased bacterial load of the hospital patients ("source") and immunocompromised status of patients ("receivers"). The total number of contacts is likely to be greater in the community, however each contact is likely to have a substantially lower chance of successful bacterial transfer. This transmission parameter is difficult to estimate. Here the estimates for the case study fitted these transmission rates to give the carriage prevalence for cephalosporin-resistant *E. coli* seen in England [4, 5] (Table 1).

It is assumed that there is a certain rate (i_c, i_h) at which those carrying bacteria become infected, which differs by setting. Due to potential fitness costs to resistant strains, it is assumed that resistant strains are equally or less likely to cause an infection than susceptible strains (by a factor r_{inf}) (Table 1). For our case study, the incidence of *E. coli* bacteraemias at 64·1 cases per 100,000 across 2013/15 [6] was used to calculate the infection rate (i_c) per day. A proportion of those that become infected die and those who die are replaced by a person with susceptible bacteria in the community. The mortality rate for people infected with ARB was taken to be higher than for patients with susceptible bacteria based on bacteraemia data [7].

People do not remain persistently colonised with ARB in the community, but instead carriage is lost (at a rate c). Due to the short duration of stay, and the likely higher transmission rates in hospital, it was assumed that resistant bacterial carriage is not lost in the hospital setting.

1.3 Model simulation

The deterministic model was constructed in R [8]. The initial population of 100,000 individuals was split into the correct proportions in hospital vs. community with zero prevalence of resistance, but everyone colonised with susceptible bacteria. The model was then run until an equilibrium was reached, and then the proportion of all people with resistant bacteria that had acquired it in the hospital was calculated (1).

Of the 10,000 LHS parameter samples, 3438 gave negative popuation sizes or invalid outputs. This would be expected with the wide ranges given for the parameters - some extreme combinations would give unreal dynamics. This left us with a "valid" set of 6562 parameter sets.

2 Parameter calculations

Nh: The hospital population: The percentage of the total English population in hospital was calculated as the total number of overnight beds in England in 2015 (130,000) [9] divided by population (53 million) to give $0{\cdot}25\%$. A range for this parameter of between $0{\cdot}02\%$ and 3% was explored. This was achieved by varying the exit and entry rates.

α: The rate at which those in the community enter the hospital was calculated from the data which states that there were 16 million admissions in England in a year (2014) [10] and the English population is 53

million. So a per day probability of entry rate was 16 / 53 / 365. This was varied between 4 million and 40 million admissions a day.

l: The rate at which those in the hospital return to the community is the inverse of the expected length of stay. In order to maintain the fixed $0{\cdot}25\%$ of the population in hospital (Nh), this parameter was varied until this percentage remained fixed at the baseline incoming from the community (α) rate. This gave a mean length of stay of $3{\cdot}15$ days. This was varied to give differing exit rates and hence numbers in hospital (Nh).

ϵ: The proportion that acquire resistance during treatment is a complex parameter to estimate. One large review paper (from over 20 years ago), considered 173 studies of 8 antibiotic classes and 225 individual treatment regimens to find that resistance arose in $5{\cdot}6\%$ of all treatments [11]. However, it has been seen to be lower ($0{\cdot}8\%$) for acquired drug resistance in initially pan-sensitive *Mycobacterium tuberculosis* strains [12]. Other studies report a range for different antibiotics and bacterial species with an overall incidence of $1{\cdot}9\%$ [13] or a range from $4{\cdot}7\%$ to 13% [14]. In our case study, we are considering resistance to third-generation cephalosporins (which commonly reflects production of extended-spectrum β-lactamases (ESBLs)), and which typically arise via transmission, i.e. ESBL-mediated resistance does not normally arise during treatment. This suggests that the level of appearance will be low. For our case study we take the mean of the lowest estimates ($0{\cdot}8\%$ and $1{\cdot}9\%$) and set the proportion to be $1{\cdot}35\%$. The full range from $0{\cdot}8\%$ to 13% is explored in the parameter variation.

ω_c, ω_h: To calculate the rate of antibiotic exposure for β-lactams we took the data from the detailed ESPAUR report [1]. Here, total consumption in DDD per 1,000 inhabitants was combined for 2014 data on penicillins (10·27), cephalosporins (0·50) and carbapenems (0·08) to give the total of 10·85 DDD / 1000 inhabitants per year. Splitting this into community and hospital use gives 8·60 and 2·24 DDD / 1000 inhabitants respectively. However, this value ignores the time component and also the dose used. Instead, we used the English Point Prevalence Survey data from 2011 to calculate how many hospital patients were on a β-lactamase sensitive antibiotic [2]. This came to a total of 11,283 out of 50,778 patients surveyed, resulting in a prevalence of 22% of patients on these antibiotics.

Point prevalence survey data do not exist for the community and so the DDD data was used for the case study community estimate at 8.6 DDD / 1,000 per day. The range explored for the community was taken to be 5 to 50 DDD per 1,000 inhabitants per day. To link the community and hospital rates, we changed the community estimate (ω_c) within this range and then multiplied it by a factor to get the hospital estimate (ω_h). In the case study the ratio was approximately 26 ($0{\cdot}22/0{\cdot}0086$). In order to explore the situation if use in the hospital was lower than in the community (which may be the case for some antibiotics) the factor was set to range between $0{\cdot}5$ (i.e. $\omega_h = 0{\cdot}5 \times \omega_c$) up to 100.

β_h, β_c: Transmission rates: number of contacts per day and probability transmission of bacteria occurs in that contact in the hospital or community respectively. These were initially calibrated to give the case study values for cephalosporin-resistant bacteria. However, prevalence of carriage of cephalosporin-resistant bacteria in the community and the hospital is hard to determine. A study of faecal carriage in Birmingham found 11% of those community samples investigated from 2010 were positive for CTX-M ESBL [5]. This varied by a person's global origin from $8{\cdot}1\%$ to $22{\cdot}8\%$ for 'Europe' and 'Middle East/South Asia' respectively. These results were higher than a previous sample of 1,000 isolates from faecal samples from 2003 in York which found a prevalence of ESBL of $1{\cdot}9\%$ across community and hospital isolates [15]. The ratio of hospital to community resistance prevalence was $1{\cdot}4 : 1$. A higher ratio of resistance prevalence has been

seen in London hospitals from clinical infection isolates (approximately $3:1$) [4]. Thus the existing data is a mixture of colonisation and infection data.

We took the data from the most recent carriage study [5], and the ratio found between community and hospital clinical isolates, to give a prevalence of with ESBL in the hospital (3×11) of 33% assuming a community level of 11%. The community carriage rate is however based on only one study that may not be representative of the UK in general. Similarly, taking a carriage proportion and multiplying it up to give a "with ESBL" proportion in hospitals may overestimate the proportion with ESBL in the hospital setting. However, this case study aims to just give an example.

The value for β_h when calibrated to this 33% prevalence, could not hit the community prevalence of 11% even assuming transmission in the community was the same as that in the hospital. To hit 33% in the hospital, the resulting value for β_h was 1.8, giving a community prevalence of 6%, assuming the same level of transmission in the community. We took the range for β_h from this estimate and varied it between 1 and 50. The level in the community was assumed to range from being 25 times smaller to up to being double the hospital rate.

c: The rate of clearance of resistant bacteria in the community: It is assumed that the time in hospital is much shorter than the time to clearance so there is no clearance in hospital. Again, this is a parameter for which the data is scarce, often because few follow-up studies consider patients for significant periods of time and there is the complication of any re-colonisation. One study found that the median time to clearance of highly resistant Enterobacteriaceae was 42 days and 144 days for any antimicrobial-resistant bacteria [16]. Another study, in France, found the median duration of carriage of ESBL-producing bacteria to be 6.6 months [17]. However, others have found there to be no change in resistance *E. coli* carriage prevalence 180 days after hospital discharge [18]. The mean duration of carriage with multiply resistant *Klebsiella pneumoniae* was 160 days in residents of nursing or residential homes [19]. Thus the range for this parameter is explored from 42 days [16] to a maximum of 2 years (to incorporate the no change by 180 days in [18]). For those with data on a mean time to clearance, the average was 127 days (42, 179 and 160) and so this is taken for the case study situation.

i_c: The rate of infection in the community for the case study is taken as the incidence in the mandatory surveillance data on *E. coli* bacteraemias: 64.1 cases per 100,000 across 2013/15 [6]. This results in a value for our case study of $64{\cdot}1/100,000/365$ which is $1{\cdot}75 \times 10^{-6}$. This is a rate and so can be applied in the hospital. However due to the co-morbidities of patients in the hospital, which will increase the risk of infection, the rate is taken to be higher in hospitals. A factor of 100 for the case study was assumed. This parameter is conservative in that there would also be other infections, more than just bacteraemias, and so an underestimate of the total number but is unlikely to affect our ratio of selection between hospital and community.

r_{inf} The decreased rate of infection for resistant strains is taken due to the fact that most resistant bacteria have been found to have a fitness cost [20] at least initially. However recent work suggests that it could be ameliorated [21] and hence a range of up to 1, or equal likelihood of infection was explored.

μ_r, μ_s A wide range of differences in the impact of resistance on mortality has been found with relative risks ranging between 1 (no impact) (e.g. [**?**]) and up to 3 [**? ?**]. For the case study, we took data from a UK study ([7]) that gave relative mortality rates for infections caused by ESBL-producing bacteria from a big enough sample of patients to detect a difference [**?**]. Our range of values explored reflect the above

mentioned variability found across the studies from different hospital and geographic settings.

3 Additional results

3.1 Proportion of the population in hospital

Corresponding to the changing exit rate and incoming hospital rate in the main text Figure 2c, the changing proportion of patients in the hospital is plotted in Figure 1. The range is from $0{\cdot}02\%$ to 3%. Comparing this to Figure 2c in the main text it can be seen that as the percentage in hospital increases (towards the bottom right corner of the graph Figure 1), the proportion of resistance acquired in hospitals also increases (towards the bottom right corner of Figure 2c in the main text).

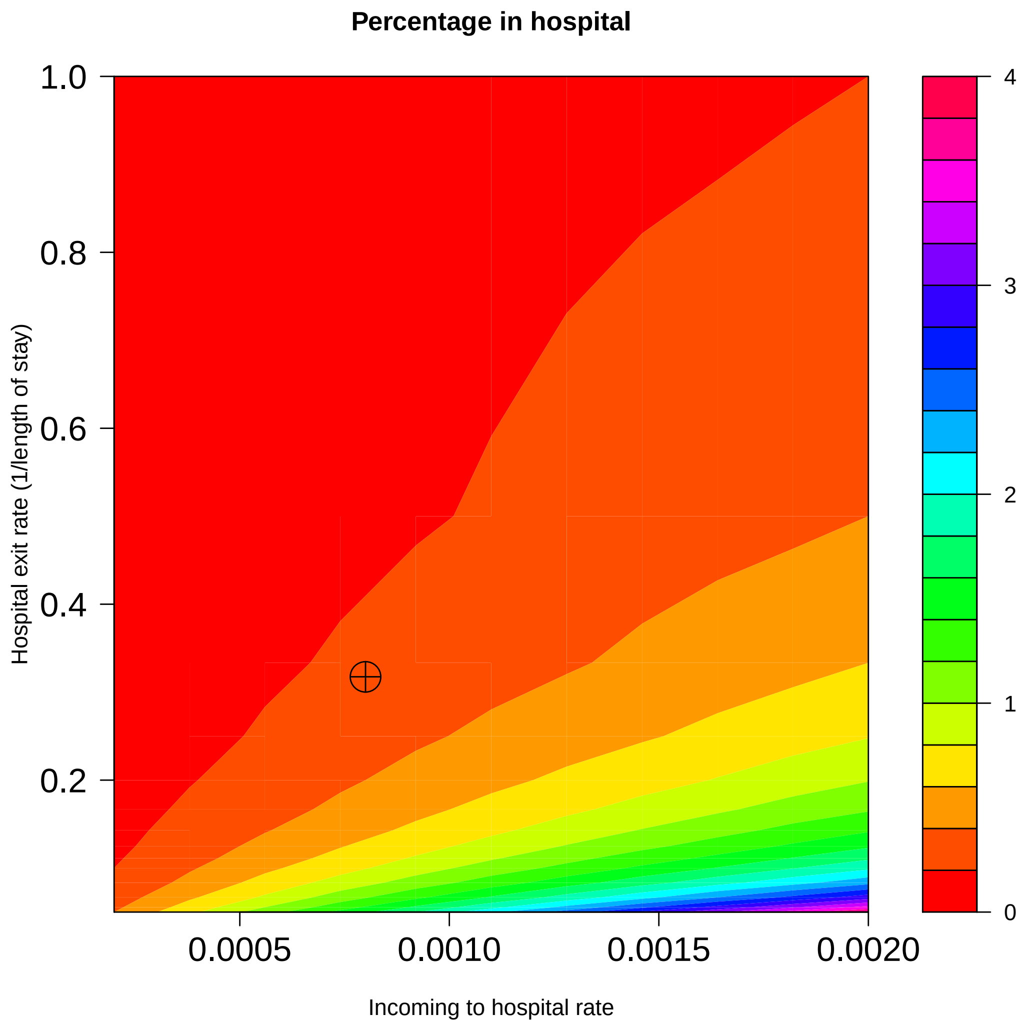

Figure 1: The percentage of the population in hospital as the exit and entry rate varies.

At 1% or 2% of the population being in our hospital setting, a mean of 34% and 38% of ARB in the total population were acquired in hospital.

3.2 Variation in infection and mortality rates

Additional results figures for the proportion of antibiotic resistance selected for in the community with variation in infection and mortality rates are given in Figure 2. This shows that varying the infection or mortality rates between hospital and the community had very little impact on the proportion on the total population with resistance that acquired it in the hospital (Figure 2) and that this was the minority as for the other parameter combinations.

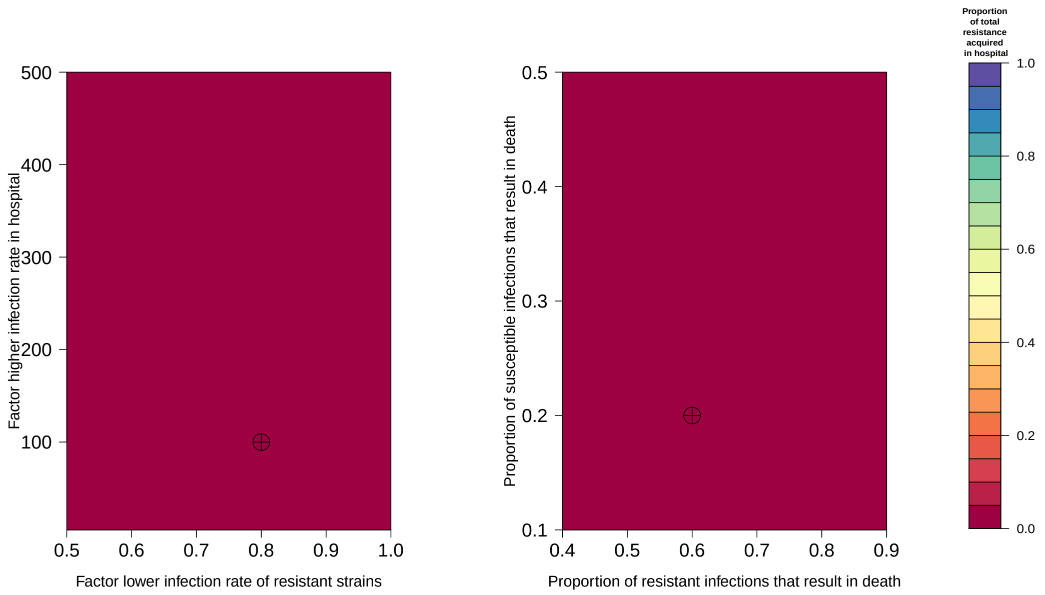

(a) (b)

Figure 2: Under our parameter explorations, the majority of people with resistance acquired the strain in the community. Here, the proportion of the population with resistance that was acquired in the hospital at different parameter levels is shown with red colours indicating that the minority acquired resistance in the hospital setting. The dashed lines indicate the boundary of 50% resistance selected for in the hospital, with blue/green levels indicating the majority of acquisition was in the hospital setting with (a) varying infection and (b) mortality rates. The target indicates the parameter combinations in our case study.

3.3 Histogram of parameter sets

A histogram plot for the proportion of total ARB selected in hospital for the total and hospital populations for the 6,562 valid parameter show a highly skewed nature (Figure 3). For the total population the distribution was positively skewed with a mean of 31%, median of 26% and lower/ upper quartiles of 10% and 49% respectively of ARB acquired in hospitals. For the hospital popuation, the density distribution was negatively skewed with a mean of 71%, median of 80% and lower/ upper quartiles of 54% and 92% respectively of ARB acquired in hospitals.

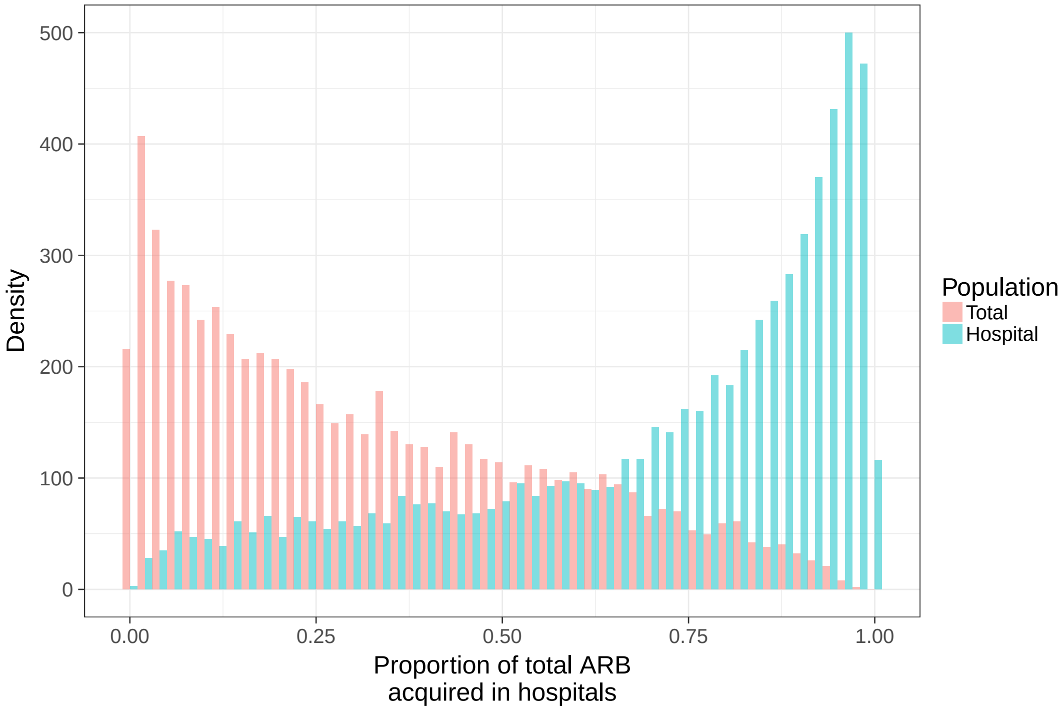

Figure 3: Density plot for the proportion of total ARB selected for in hospitals from the valid LHS parameter combinations

3.4 Sensitivity analysis for hospital population

In a sensitivity analysis of the proportion of acquisition in hospital that occurs in hospital, we found that the most influential parameters are those of antibiotic exposure, rate of clearance of resistance in the community and length of stay (hospital exit rate) (Figure 4).

3.5 Sensitivity analysis for resistance prevalence

If we consider the impact of parameter variation on the prevalence of resistance we find a similar set of parameters to be important (Figure 5). However, the most correlated was rate of clearance of resistance in the community.

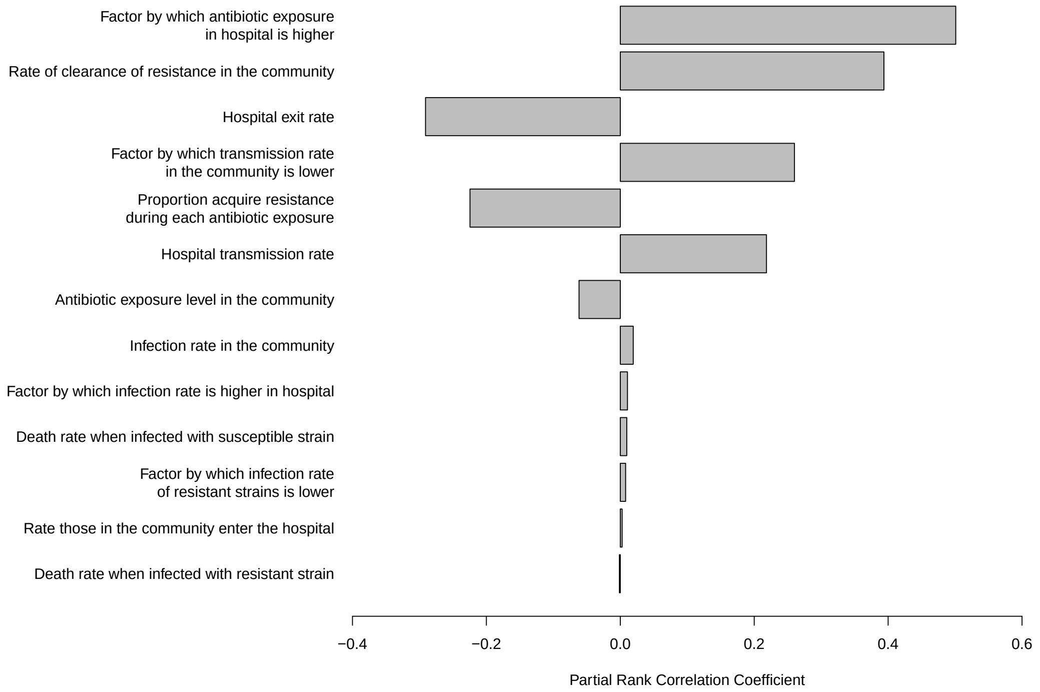

Figure 4: The rate at which resistance is cleared in the community and antibiotic exposure level in the community are the key drivers of acquisition in hospital for the hospital population. This is shown by them ranking the highest in this Partial Rank Correlation Coefficient analysis of key parameters against proportion of resistance in hospital selected for in hospital.

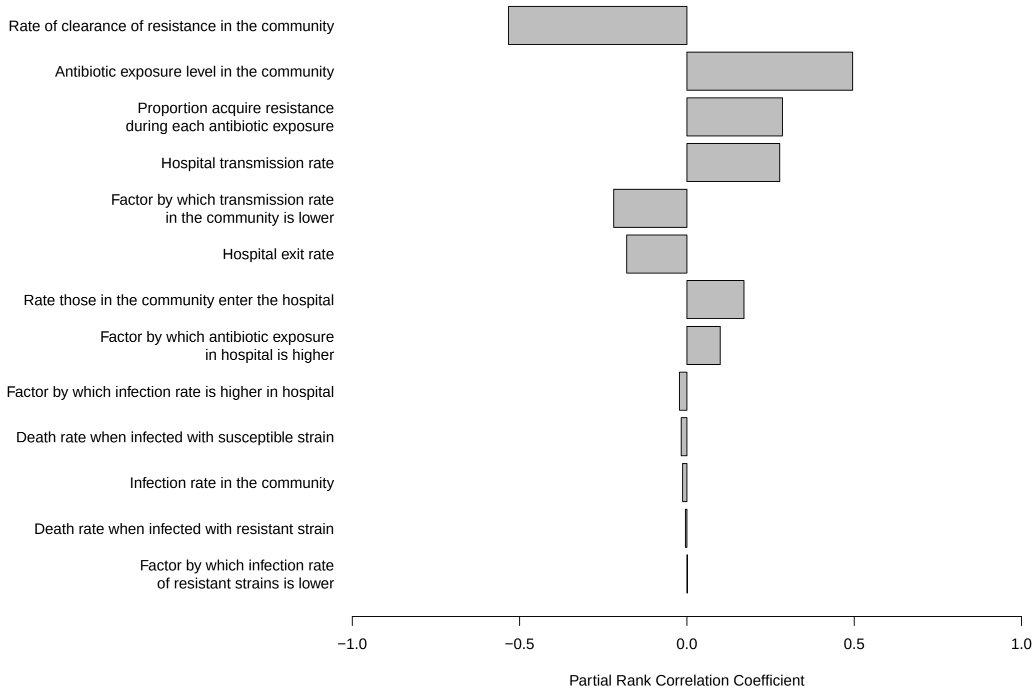

Figure 5: Tornado diagram of the key drivers of resistance prevalence from Partial Rank Correlation Coefficient analysis. The ranking is in term of their PRCC value against final total prevalence of resistance carriage per 100,000.

References

[1] Public Health England. English Surveillance programme for Antimicrobial utilisation and Resistance (ESPAUR) 2010 to 2014: Report 2015, 2015. URL https://www.gov.uk/government/uploads/system/uploads/attachment_data/file/477962/ESPAUR_Report_2015.pdf.

[2] Health Protection Agency. English National Point Prevalence Survey on Healthcare-associated Infections and Antimicrobial Use, 2011: Preliminary data., 2012. URL https://www.gov.uk/government/uploads/system/uploads/attachment_data/file/331871/English_National_Point_Prevalence_Survey_on_Healthcare_associated_Infections_and_Antimicrobial_Use_2011.pdf.

[3] B. S. Cooper, G. F. Medley, S. P. Stone, C. C. Kibbler, B. D. Cookson, J. A. Roberts, G. Duckworth, R. Lai, and S. Ebrahim. Methicillin-resistant Staphylococcus aureus in hospitals and the community: stealth dynamics and control catastrophes. *Proc Natl Acad Sci U S A*, 101(27):10223–10228, Jul 2004. doi: 10.1073/pnas.0401324101. URL http://dx.doi.org/10.1073/pnas.0401324101.

[4] L. S. Moore, R. Freeman, M. J. Gilchrist, M. Gharbi, E. T. Brannigan, H. Donaldson, D. M. Livermore, and A. H. Holmes. Homogeneity of antimicrobial policy, yet heterogeneity of antimicrobial resistance: antimicrobial non-susceptibility among 108,717 clinical isolates from primary, secondary and tertiary care patients in London. *J Antimicrob Chemother*, 69(12):3409–22, 2014. doi: 10.1093/jac/dku307. URL http://www.ncbi.nlm.nih.gov/pubmed/25118270.

[5] Nimal H. Wickramasinghe, Li Xu, Andrew Eustace, Sahida Shabir, Tranprit Saluja, and Peter M. Hawkey. High community faecal carriage rates of CTX-M ESBL-producing Escherichia coli in a specific population group in Birmingham, UK. *J Antimicrob Chemother*, 67(5):1108–1113, May 2012. doi: 10.1093/jac/dks018. URL http://dx.doi.org/10.1093/jac/dks018.

[6] PHE. Annual Epidemiological Commentary:Mandatory MRSA, MSSA and E. coli bacteraemia and C. difficile infection data, 2013/14. Technical report, 2014. URL https://www.gov.uk/government/uploads/system/uploads/attachment_data/file/330529/HCAI_mandatory_surveillance_annual_epidemiological_commentary_2013_14.pdf.

[7] Mark Melzer and Irene Petersen. Mortality following bacteraemic infection caused by extended spectrum beta-lactamase (ESBL) producing E. coli compared to non-ESBL producing E. coli. *J Infect*, 55 (3):254–259, Sep 2007. doi: 10.1016/j.jinf.2007.04.007. URL http://dx.doi.org/10.1016/j.jinf.2007.04.007.

[8] R Core Team. R: A Language and Environment, 2005.

[9] NHS England. Average number of available and occupied beds open overnight by sector, November 2015. URL https://www.england.nhs.uk/statistics/statistical-work-areas/bed-availability-and-occupancy/bed-data-overnight/.

[10] Health and Social Care Information Centre. Hostpial Episode Statistics: Admitted Patient Care, England - 2014-2015, November 2015. URL http://www.hscic.gov.uk/catalogue/PUB19124/hosp-epis-stat-admi-summ-rep-2014-15-rep.pdf.

[11] Douglas N Fish, Stephen C Piscitelli, and Larry H Danziger. Development of resistance during antimicrobial therapy: a review of antibiotic classes and patient characteristics in 173 studies. *Pharmacotherapy: The Journal of Human Pharmacology and Drug Therapy*, 15(3):279–291, 1995.

[12] W. Lew, M. Pai, O. Oxlade, D. Martin, and D. Menzies. Initial drug resistance and tuberculosis treatment outcomes: systematic review and meta-analysis. *Ann Intern Med*, 149(2):123–34, 2008. Lew, Woojin Pai, Madhukar Oxlade, Olivia Martin, Daniel Menzies, Dick Ann Intern Med. 2008 Jul 15;149(2):123-34.

[13] Sang-Ho Choi, Jung Eun Lee, Su Jin Park, Seong-Ho Choi, Sang-Oh Lee, Jin-Yong Jeong, Mi-Na Kim, Jun Hee Woo, and Yang Soo Kim. Emergence of antibiotic resistance during therapy for infections caused by Enterobacteriaceae producing AmpC β-lactamase: implications for antibiotic use. *Antimicrobial agents and chemotherapy*, 52(3):995–1000, 2008.

[14] D Milatovic and I Braveny. Development of resistance during antibiotic therapy. *European journal of clinical microbiology*, 6(3):234–244, 1987.

[15] C. J. Munday, G. M. Whitehead, N. J. Todd, M. Campbell, and P. M. Hawkey. Predominance and genetic diversity of community- and hospital-acquired CTX-M extended-spectrum beta-lactamases in York, UK. *J Antimicrob Chemother*, 54(3):628–633, Sep 2004. doi: 10.1093/jac/dkh397. URL http://dx.doi.org/10.1093/jac/dkh397.

[16] Manon R. Haverkate, Lennie P G. Derde, Christian Brun-Buisson, Marc J M. Bonten, and Martin C J. Bootsma. Duration of colonization with antimicrobial-resistant bacteria after ICU discharge. *Intensive Care Med*, 40(4):564–571, Apr 2014. doi: 10.1007/s00134-014-3225-8. URL http://dx.doi.org/10.1007/s00134-014-3225-8.

[17] Gabriel Birgand, Laurence Armand-Lefevre, Isabelle Lolom, Etienne Ruppe, Antoine Andremont, and Jean-Christophe Lucet. Duration of colonization by extended-spectrum beta-lactamase-producing Enterobacteriaceae after hospital discharge. *American journal of infection control*, 41(5):443–447, 2013.

[18] N Bruinsma, PMG Filius, AE Van Den Bogaard, Sita Nys, J Degener, H Ph Endtz, and EE Stobberingh. Hospitalization, a risk factor for antibiotic-resistant Escherichia coli in the community? *Journal of Antimicrobial Chemotherapy*, 51(4):1029–1032, 2003.

[19] J Bird, R Browning, RP Hobson, FM MacKenzie, J Brand, and IM Gould. Multiply-resistant Klebsiella pneumoniae: failure of spread in community-based elderly care facilities. *Journal of Hospital Infection*, 40(3):243–247, 1998.

[20] Dan I. Andersson and Diarmaid Hughes. Antibiotic resistance and its cost: is it possible to reverse resistance? *Nat Rev Microbiol*, 8(4):260–271, Apr 2010. doi: 10.1038/nrmicro2319. URL http://dx.doi.org/10.1038/nrmicro2319.

[21] Damien Roux, Olga Danilchanka, Thomas Guillard, Vincent Cattoir, Hugues Aschard, Yang Fu, Francois Angoulvant, Jonathan Messika, Jean-Damien Ricard, John J. Mekalanos, Stephen Lory, Gerald B. Pier, and David Skurnik. Fitness cost of antibiotic susceptibility during bacterial infection. *Sci Transl Med*, 7(297):297ra114, Jul 2015. doi: 10.1126/scitranslmed.aab1621. URL http://dx.doi.org/10.1126/scitranslmed.aab1621.
